# Ropivacaine attenuates endotoxin plus hyperinflation-mediated acute lung injury via inhibition of early-onset Src-dependent signaling

**DOI:** 10.1186/1471-2253-14-57

**Published:** 2014-07-19

**Authors:** Tobias Piegeler, Randal O Dull, Guochang Hu, Maricela Castellon, Andreia Z Chignalia, Ruben G Koshy, E Gina Votta-Velis, Alain Borgeat, David E Schwartz, Beatrice Beck-Schimmer, Richard D Minshall

**Affiliations:** 1 Department of Anesthesiology, University of Illinois Hospital > Health Sciences System, 835 S. Wolcott Ave (m/c 868), Chicago, IL 60612, USA; 2 Department of Pharmacology, University of Illinois Hospital > Health Sciences System, Chicago, IL, USA; 3 Department of Bioengineering, University of Illinois Hospital > Health Sciences System, Chicago, IL, USA; 4 Center for Lung and Vascular Biology, University of Illinois Hospital > Health Sciences System, Chicago, IL, USA; 5 Institute of Anesthesiology, University Hospital Zurich, Zurich, Switzerland; 6 Department of Anesthesiology, Balgrist University Hospital, Zurich, Switzerland; 7 Department of Anesthesiology, Jesse Brown VA Medical Center, Chicago, IL, USA

**Keywords:** Acute lung injury, ARDS, Ventilator-induced lung injury, Local anesthetics, Endothelium, Caveolin-1, Src protein tyrosine kinase

## Abstract

**Background:**

Acute lung injury (ALI) is associated with high mortality due to the lack of effective therapeutic strategies. Mechanical ventilation itself can cause ventilator-induced lung injury. Pulmonary vascular barrier function, regulated in part by Src kinase-dependent phosphorylation of caveolin-1 and intercellular adhesion molecule-1 (ICAM-1), plays a crucial role in the development of protein-/neutrophil-rich pulmonary edema, the hallmark of ALI. Amide-linked local anesthetics, such as ropivacaine, have anti-inflammatory properties in experimental ALI. We hypothesized ropivacaine may attenuate inflammation in a “double-hit” model of ALI triggered by bacterial endotoxin plus hyperinflation via inhibition of Src-dependent signaling.

**Methods:**

C57BL/6 (WT) and *ICAM-1*^*−/−*^ mice were exposed to either nebulized normal saline (NS) or lipopolysaccharide (LPS, 10 mg) for 1 hour. An intravenous bolus of 0.33 mg/kg ropivacaine or vehicle was followed by mechanical ventilation with normal (7 ml/kg, NTV) or high tidal volume (28 ml/kg, HTV) for 2 hours. Measures of ALI (excess lung water (ELW), extravascular plasma equivalents, permeability index, myeloperoxidase activity) were assessed and lungs were homogenized for Western blot analysis of phosphorylated and total Src, ICAM-1 and caveolin-1. Additional experiments evaluated effects of ropivacaine on LPS-induced phosphorylation/expression of Src, ICAM-1 and caveolin-1 in human lung microvascular endothelial cells (HLMVEC).

**Results:**

WT mice treated with LPS alone showed a 49% increase in ELW compared to control animals (*p* = 0.001), which was attenuated by ropivacaine (*p* = 0.001). HTV ventilation alone increased measures of ALI even more than LPS, an effect which was not altered by ropivacaine. LPS plus hyperinflation (“double-hit”) increased all ALI parameters (ELW, EVPE, permeability index, MPO activity) by 3–4 fold compared to control, which were again decreased by ropivacaine. Western blot analyses of lung homogenates as well as HLMVEC treated in culture with LPS alone showed a reduction in Src activation/expression, as well as ICAM-1 expression and caveolin-1 phosphorylation. In *ICAM-1*^*−/−*^ mice, neither addition of LPS to HTV ventilation alone nor ropivacaine had an effect on the development of ALI.

**Conclusions:**

Ropivacaine may be a promising therapeutic agent for treating the cause of pulmonary edema by blocking inflammatory Src signaling, ICAM-1 expression, leukocyte infiltration, and vascular hyperpermeability.

## Background

Acute lung injury (ALI) and acute respiratory distress syndrome (ARDS) are associated with high morbidity and mortality [1,2]. Treatment options are largely supportive (e.g. lung-protective ventilation) [3] and thus there is a need for effective anti-inflammatory strategies that treat pulmonary endothelial hyperpermeability, a hallmark of the disease. Despite the necessity of mechanical ventilation in the supportive management of ALI or ARDS, ventilation *per se* can also contribute to lung injury (i.e., ventilator-induced lung injury, or VILI) [4]. Large tidal volumes, high inspiratory pressures, and/or high levels of positive end-expiratory pressure lead to a protein- and neutrophil-rich permeability-type pulmonary edema, similar to that occurring in ALI [5]. Experimental data also suggests that VILI is even more severe when induced in combination with bacterial endotoxin, as exemplified in the “double-hit” mouse model of ALI/VILI [6].

Local anesthetics are widely used in clinical practice for local, regional and neuraxial anesthesia, as well as for peri- and postoperative pain control [7,8]. In addition, they have also been demonstrated to exhibit significant anti-inflammatory properties [9-11]. In an earlier study of bacterial endotoxin-induced lung injury, it was shown that administration of the long-acting amide-linked local anesthetic ropivacaine attenuated endothelial cell NFκB activation and inflammatory lung injury *in vivo* and lung epithelial cell activation *in vitro*[9].

Src protein tyrosine kinase (Src) is known to regulate endothelial barrier function and to play a key role in mediating inflammatory vascular hyperpermeability *in vitro*[12]. Intercellular adhesion molecule-1 (ICAM-1), a cell surface molecule of the immunoglobulin superfamily, mediates firm adhesion of circulating neutrophils and thereby enhances their transmigration through the endothelial barrier. ICAM-1 is phosphorylated by Src on tyrosine 512 (human; tyrosine 518 in mice) [13,14] during pulmonary vascular inflammation and is thought to play an important role in the mechanism of ALI and ARDS [14-16]. Additionally, Src-dependent phosphorylation of caveolin-1 on tyrosine 14, which is known to promote the transcellular transport of albumin via caveolae [17] as well as regulate downstream signaling that can lead to disruption of adherens junctions [18], also contributes significantly to the development of ALI [12,19,20].

In the present study, we hypothesized that the local anesthetic ropivacaine, at clinically relevant concentrations, may have anti-inflammatory properties by blocking endotoxin-induced Src signaling in mice and cultured human endothelial cells. We tested the hypothesis that ropivacaine attenuates lung injury/inflammatory hyper-permeability in the mouse induced by either high tidal volume (HTV) ventilation or bacterial endotoxin alone, versus the “double-hit” model of HTV ventilation together with bacterial endotoxin [6]. In these experiments, we assessed whether anti-inflammatory effects were mediated by reducing Src-dependent phosphorylation of ICAM-1 and caveolin-1 in endothelial cells, thereby blocking inflammatory hyper-permeability [12].

## Methods

### Animals

All animal experiments were approved by the University of Illinois Institutional Animal Care and Use Committee (IACUC). Mice were housed in the mouse barrier facility at UIC and were allowed full access to food and water until the time of the experiment. Two strains of mice were used in this study, wild-type C57BL/6 (*WT*) and strain-matched ICAM-1 knockout (*ICAM-1*^*−/−*^) mice.

### Animal protocol

**Figure 1:**
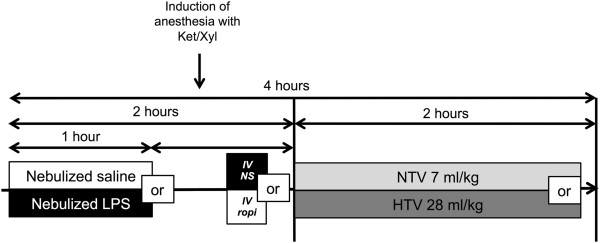
**Schematic illustration of the workflow of animal experiments.** C57BL/6 mice (*WT)* were exposed to either normal saline (NS) or nebulized LPS (10 mg) for 1 hour. Induction of anesthesia with ketamine/xylazine (Ket/Xyl) during the second hour of the experiment was followed by the insertion of a central venous catheter into the right or left internal jugular vein and the application of an intravenous bolus of either 0.01 mg ropivacaine (ropi in the figure, R in the group description) or vehicle (normal saline, NS). Mice were then mechanically ventilated for 2 hours via a tracheostomy with either normal tidal volumes (7 ml/kg, NTV) or high tidal volumes (28 ml/kg, HTV). Mice were randomized into 8 different groups (n = 7 each): 1) Nebulized NS, intravenous NS, NTV ventilation (NS-NS-NTV; control), 2) NS-R-NTV, 3) LPS-NS-NTV, 4) LPS-R-NTV, 5) NS-NS-HTV, 6) NS-R-HTV, 7) LPS-NS-HTV, 8) LPS-R-HTV.

To evaluate the effects of ropivacaine on acute lung injury as induced by high tidal volume ventilation or endotoxin, as well as by the “double-hit” with endotoxin and VILI, mice were exposed to either nebulized normal saline (NS, 0.9% sodium chloride solution, Hospira, Lake Forest, IL) or 10 mg nebulized *Escherichia coli* serotype 055:B5 lipopolysaccharide diluted in NS (LPS, Sigma-Aldrich, St. Louis, MO) for 1 hour. During the second hour of the protocol, anesthesia was induced via intraperitoneal injection of 100 mg/kg ketamine (Hospira) and 5 mg/kg xylazine (Lloyd Laboratories, Shenandoah, IA) and a PE10 catheter was inserted into the right internal jugular vein. Two hours after the start of the experiment, mice received a 30 μl intravenous bolus of either NS or ropivacaine (R) at 1 mM concentration (Naropin®, APP Pharmaceuticals, Schaumburg, IL) diluted in NS solution. The total amount of administered drug was therefore 0.01 mg, or 0.33 mg/kg in a 30 g mouse. Mice were then subjected to volume-controlled mechanical ventilation via a tracheostomy with either a normal tidal volume of 7 ml/kg (NTV) or high tidal volume of 28 ml/kg (HTV) to induce VILI [21-23]. Mice were randomly assigned to one of eight groups (n = 7 each) as shown in Figure 1: 1) Nebulized NS, intravenous NS, NTV ventilation (NS-NS-NTV; control), 2) NS-R-NTV, 3) LPS-NS-NTV, 4) LPS-R-NTV, 5) NS-NS-HTV, 6) NS-R-HTV, 7) LPS-NS-HTV, 8) LPS-R-HTV. Mice were given an additional intraperitoneal ketamine/xylazine injection (half of initial dose) 1 hour after the initiation of mechanical ventilation to maintain anesthesia. Normothermia (37°C to 38°C) was maintained using a heating lamp.

### Excess lung water (ELW), extravascular plasma equivalents (EVPE), permeability index

One hour before the end of the experiment, mice received an intravenous injection of radioactive-labeled albumin (1 μC I^125^-albumin). At the end of the experiment, the body weight of the animal was determined and a blood sample was collected, either via a retro-orbital approach or by puncturing the inferior vena cava after opening up the abdominal cavity, and hematocrit was determined (IEC MB Centrifuge, Damon, Needham Heights, MA). An additional 400 – 500 μl of blood was collected in tubes containing ethylenediaminetetraacetic acid (EDTA; BD Biosciences, Franklin Lakes, NJ). The lungs were then excised in total, placed into pre-weighed 5 ml plastic tubes (BD Biosciences) and immediately covered to prevent evaporation. After initial weighing of the tube together with the lung, we added 1 ml of ultrapure water and re-weighed the containers. The lungs were then homogenized with a Kinematica Polytron homogenizer (Fisher Scientific, Pittsburgh, PA). A 0.25 ml aliquot of lung homogenate was sedimented at 13000 rpm for 10 min (Eppendorf 5415R microcentrifuge, Eppendorf, Hamburg, Germany). Hemoglobin concentration was measured in both the supernatant of the homogenate and the anti-coagulated whole blood sample with a Hb 201 analyzer (Hemocue, Cypress, CA). Total lung homogenate (150 μl, before centrifugation), supernatant, and a whole blood sample were then put in pre-weighed aluminum dishes and weighed. The dishes were then placed in a drying oven (60°C) for at least 24 hours, after which the dried samples were weighed again.

ELW was then calculated as described by Su et al. [24]:

First, we calculated the water fraction in the lung homogenate (WF_h_), the supernatant of the homogenate (WF_s_) and the whole blood sample (WF_b_) using the formula

$$\mathrm{WF}\;=\;\left(W_{\mathrm{wet}}–\;W_{\mathrm{dry}}\right)\;/\;W_{\mathrm{wet}},$$

with W_wet_ representing the wet weight and W_dry_ being the dry weight.

We then calculated the blood volume found in the lung:

$$Q_{b}=\;1.039\;x\;\left(Q_{h}x\;WF_{h}x\;Hb_{s}\right)\;/\;\left(WF_{s}x\;Hb_{b}\right)$$

The number of 1.039 is the density of blood, Q_h_ denotes the lung homogenate weight (=whole lung wet weight + weight of ultrapure water), Hb_s_ represents the hemoglobin concentration in the homogenate supernatant, and Hb_b_ the hemoglobin concentration of the whole blood sample.

The calculation of the volume of water in the lung was executed with the following formula:

$$Q_{w}=\;\left(Q_{h}x\;WF_{h}\right)\;–\;\left(Q_{b}x\;WF_{b}\right)\;–\;W_{w}.$$

Here, W_w_ represents the weight of the ultrapure water added before the homogenization of lung tissue (see above).

The calculation of the whole lung dry weight (Q_d_) was also necessary:

$$Q_{d}=\;\mathrm{whole}\;\mathrm{lung}\;\mathrm{wet}\;\mathrm{weight}\;–\;Q_{w}–\;Q_{b}$$

Finally, ELW was calculated as follows:

$$\begin{array}{ll} \mathrm{ELW}\;= & \;\left\{\left(Q_{w\;\exp}/\;Q_{d\;\exp}x\;{Q_{d}}_{\exp}\right)\;x\;\left(Q_{w\;\mathrm{control}}/\;Q_{d\;\mathrm{control}}x\;Q_{d\;\mathrm{control}}\right)\right\}\\ & \;x\;1000\;\left(\mathrm{\mu l}\right). \end{array}$$

Q_w exp_ is the volume of water found in the lung of experimental animals and Q_d exp_ is the dry weight of the lung in the experimental animals. Mice of the same age as the experimental groups served as controls.

For EVPE, we additionally assessed radioactivity of both 100 μl whole blood and the whole lung immersed in 1 ml of water before homogenization in a γ-counter chamber (Perkin Elmer, Waltham, MA). Measured counts per minute were then normalized per gram (cpg) and used in the formula to determine EVPE as follows:

$$\mathrm{EVPE}\;=\;\left[\mathrm{blood}\;\mathrm{free}\;\mathrm{lung}\;\mathrm{tissue}\;\mathrm{cpg}\;/\left(\mathrm{plasma}\;\mathrm{cpg}\;x\;1000\right)\right].$$

For the calculation of the endothelial permeability index, we first calculated the plasma volume:

$$\mathrm{Plasma}\;\mathrm{Volume}\;=\;\left[\left(\mathrm{body}\;\mathrm{weight}\right)\;x\;\left(1\;–\;\mathrm{Hematocrit}\right)\;x\;0.007\;x\;1000\right].$$

The results from this calculation were used to determine the permeability index:

$$\begin{array}{ll} \mathrm{Endothelial}\;\mathrm{permeability}= & \;\left[\mathrm{blood}-\mathrm{free}\;\mathrm{lung}\;\mathrm{tissue}\;\mathrm{cpg}\right)\\ & \left(\;/\left(\mathrm{plasma}\;\mathrm{cpg}\;x\;\mathrm{plasma}\;\mathrm{volume}\right)\right]\\ & x\;100\;\left(\%\right). \end{array}$$

### Myeloperoxidase activity

Myeloperoxidase (MPO) activity in lung homogenates was used as a surrogate marker of neutrophil extravasation and sequestration as previously described [6,25].

### Lung homogenates for Western blot analysis

Whole lungs of animals from the different treatment groups were removed at the end of the experiment, immediately snap-frozen in liquid nitrogen, and stored at −80°C until further processing. Equal lung parts were then immersed in lysis buffer (0.4 M Tris–HCl pH 7.5, 0.14 M NaCl, 1.5% Triton X-100, 0.5% desoxycholic acid, 0.1% sodium dodecyl sulfate, protease inhibitor cocktail, 200 mM phenylmethylsulfonylfluoride, 1 mM EDTA, 1 mM sodium-fluoride, and 1 mM sodium-orthovanadate) and homogenized on ice. After a 30-minute incubation to allow protein dissociation, the homogenates were centrifuged for 20 minutes at 16,000 rpm to remove debris. The supernatant was collected and stored at −80°C until further use.

### Cell cultures and in vitro experimental procedures

Human lung microvascular endothelial cells (HLMVEC, Lonza, Walkersville, MD) were cultured in VascuLife Basal medium supplemented with the VascuLife VEGF-Mv LifeFactors kit (both from LifeLine Cell Technology, Frederick, MD) containing 5% fetal bovine serum, 10 mM L-glutamine, 5 ng/ml recombinant human (rh) vascular endothelial growth factor (VEGF), 5 ng/ml rh epidermal growth factor (EGF), 5 ng/ml rh fibroblast growth factor (FGF), 15 ng/ml rh insulin-like growth factor 1 (IGF-1), 1 μg/ml hydrocortisone hemisuccinate, 0.75 U/ml heparin, and 50 μg/ml ascorbic acid. All cell culture dishes, plates and flasks were coated with 0.2% gelatin (Sigma-Aldrich, St. Louis, MO) diluted in Dulbecco’s phosphate buffered saline without calcium and magnesium (Cellgro, Manassas, VA). Experiments were performed in 90 – 100% confluent cells from passages 5 – 12. Before the experiments, cells were grown in VascuLife Basal Medium without supplements for 16 hours. Cells were kept in 5% CO_2_ and 95% room air in a water-jacketed incubator at 37°C.

HLMVEC monolayers were incubated with *Escherichia coli* serotype 055:B5 lipopolysaccharide (LPS, Sigma-Aldrich, St. Louis, MO) at a concentration of 4 μg/ml diluted in VascuLife Basal Medium for 4 hours. Ropivacaine 0.5% was also diluted in VascuLife Basal Medium in order to be able to test several concentrations of the drug (1 nM, 1 μM, 10 μM, 100 μM) in the absence or presence of LPS. For some experiments, cells were pretreated for 30 minutes with the Src kinase inhibitor 4-amino-5-(4-chlorophenyl)-7-(dimethylethyl)pyrazolo[3,4-*d*] pyrimidine (PP2; 10 μM) dissolved in dimethylsulfoxide (DMSO, Sigma-Aldrich) or with DMSO alone as the vehicle control (0.1%) prior to addition of LPS.

### Cell harvest and lysis

At the end of the experiment, HLMVEC were harvested and lysed with radio-immunoprecipitation buffer (Boston Bioproducts, Ashland, MA), supplemented with protease inhibitor cocktail, 200 mM phenylmethylsulfonylfluoride, 1 mM EDTA, 1 mM sodium-fluoride, and 1 mM sodium-orthovanadate (all from Sigma-Aldrich) as previously described [26,27].

### Determination of total protein concentration

Total protein concentration of lung homogenates and whole cell lysates was measured by DC Protein Assay Kit (BioRad, Hercules, CA) according to the manufacturer’s instructions; immunoglobulin G was used for determination of the standard curve.

### Western blot

Western blot analysis of either lung homogenates or whole cell lysates was carried out as previously described [26,27]. Antibodies against pY419 Src and total Src were purchased from Cell Signaling Technologies (Danvers, MA), for pY512 ICAM-1 and total ICAM-1 from Santa Cruz Biotechnology (Santa Cruz, CA), and for pY14 caveolin-1, total caveolin-1, and β-actin from BD Biosciences (Franklin Lakes, NJ).

### Statistical analysis

First, normal distribution was assessed using a Shapiro-Wilk test. Normally distributed data was then subject to analysis by ANOVA followed by Bonferroni *post-hoc* testing to maintain the family-wise error rate <0.05. Not-normally distributed data was analyzed with non-parametric testing methods (Kruskal-Wallis and Mann–Whitney U tests) with Dunn’s *post-hoc* correction. The *in vivo* measures of ALI (ELW, EVPE, Permeability, MPO activity) were compared as two separate sets of data: one between the groups that were subject to NTV ventilation and one between the groups that had received HTV ventilation. In both cases, the NS-NS-NTV group served as the control group. Experimental data are presented as mean ± standard deviation (SD). All tests were performed two-sided and non-blinded with GraphPad Prism for Mac, Version 6 (GraphPad Software, La Jolla, CA). A *p*-value of <0.05 was considered to be statistically significant.

## Results

### Ropivacaine attenuates measures of ALI triggered by bacterial endotoxin and hyperinflation

To assess the influence of ropivacaine administration on the degree of ALI induced by endotoxin or HTV alone vs the “double-hit” model (see Figure 1), we measured ELW, EVPE and permeability index. Additionally, the activity of neutrophil MPO in lung homogenates was evaluated as an index of lung inflammation.

**Figure 2:**
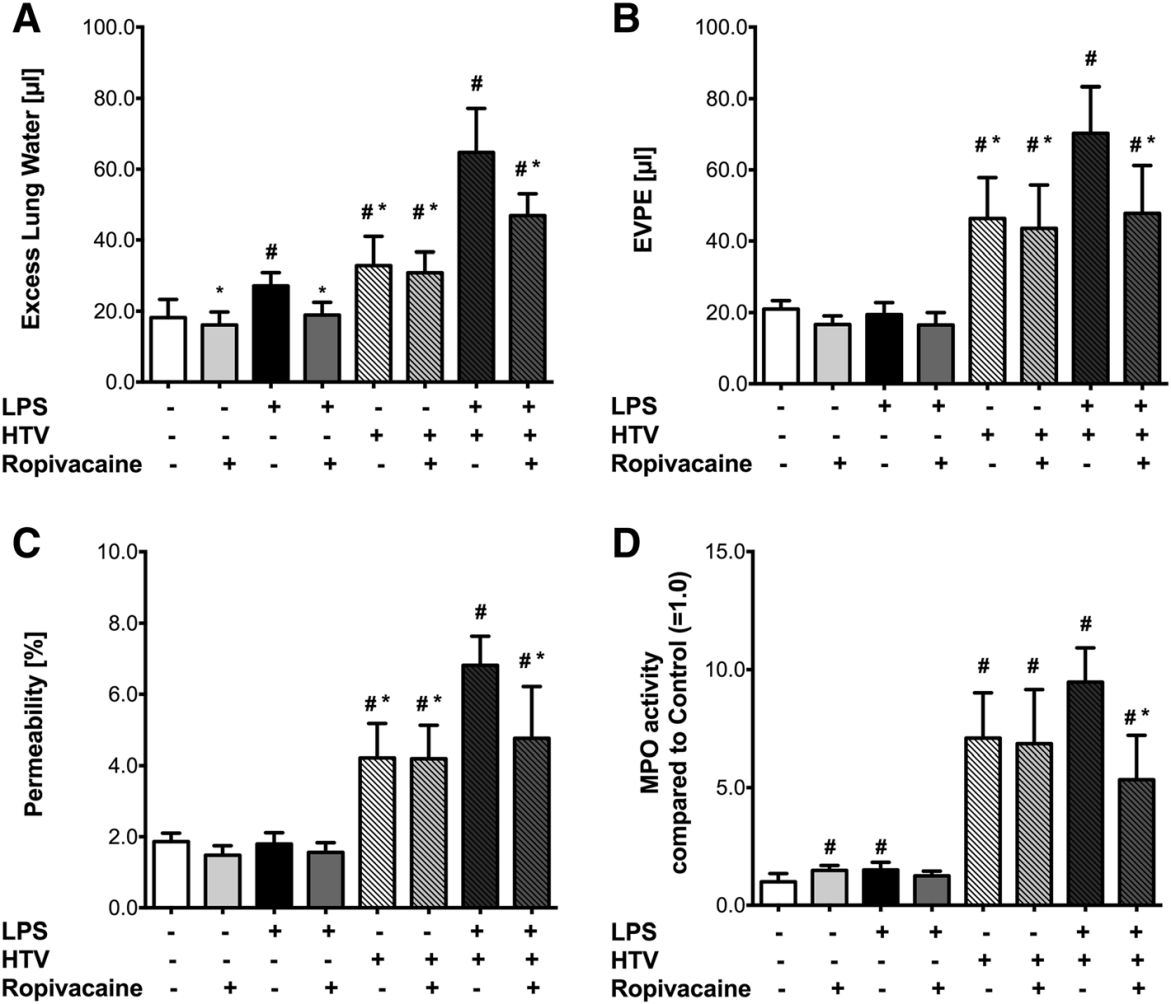
**Effect of ropivacaine on acute lung injury.** Acute lung injury was induced by either hyperinflation (HTV), bacterial lipopolysaccharide (LPS), or a combination of the two (“double-hit” model) in C57BL/6 wild type (*C57 WT*) mice. ALI was assessed by measuring **(A)** Excess lung water (ELW), **(B)** extravascular plasma equivalents (EVPE), **(C)** permeability index, and **(D)** myeloperoxidase activity as described in Methods. Values shown are mean ± SD (n = 7); ^#^*p* < 0.05 vs. control (NS-NS-HTV); *p < 0.05 vs. LPS-NS-HTV.

Treatment with LPS alone (LPS-NS-NTV) led to a 49% increase in ELW compared to control animals (NS-NS-NTV; *p* = 0.001, Figure 2A) which was significantly attenuated by ropivacaine (LPS-R-NTV group; *p* = 0.001). However, WT animals ventilated with HTV (NS-NS-HTV) also showed a significant increase in ELW compared to control animals (NS-NS-NTV; *p* = 0.005), however this effect was not altered by ropivacaine (NS-R-HTV group; *p* = 1). If mice were exposed to LPS prior to mechanical ventilation with HTV (LPS-NS-HTV), we observed a 2-fold increase in ELW compared to HTV alone (NS-NS-HTV; *p* < 0.001) and a 3.5-fold increase compared to control (NS-NS-NTV; *p* < 0.001). Ropivacaine (LPS-R-HTV group) significantly decreased the “double-hit” inflammatory response by 28% (*p* = 0.001; Figure 2A).

To further evaluate the effect of HTV ventilation, LPS, and ropivacaine on endothelial barrier function, changes in EVPE (Figure 2B) and permeability index were assessed (Figure 2C). These measures, which are based on the accumulation of radioactive ^125^I-albumin in the lungs, quantitatively assess endothelial barrier function *in vivo* during ALI. The two parameters showed patterns similar to that of ELW, except for the fact that there were no significant differences between the groups which were subject to NTV ventilation (*p* = 0.091 for EVPE; *p* = 0.113 for permeability, Figures 2B and 2C). HTV ventilation, on the other hand, induced a significant increase in EVPE and permeability compared to control WT mice (*p* = 0.006 for EVPE; *p* = 0.035 for permeability) and neither were altered by ropivacaine (*p* = 1 for both comparisons). LPS exposure prior to HTV ventilation significantly increased EVPE and permeability index compared to control (*p* < 0.001 for EVPE and permeability) and HTV ventilation alone (*p* = 0.017 for EVPE; *p* = 0.004 for permeability), and this LPS-induced increase was attenuated significantly by ropivacaine (*p* = 0.039 for EVPE; *p* = 0.042 for permeability).

To assess the amount of neutrophil transmigration into the interstitial and alveolar spaces, MPO activity in lung homogenates was measured. Here, both LPS and ropivacaine led to a slight increase in MPO activity compared to control in mice that were subsequently ventilated with NTV (*p* = 0.02 for LPS-R-NTV, *p* = 0.027 for NS-R-NTV, Figure 2D). However, there was no difference in MPO activity between control animals and those exposed to LPS and ropivacaine followed by NTV ventilation (*p* = 0.692). After HTV ventilation alone, there was a 7-fold increase in MPO activity compared to control (*p* = 0.001; Figure 2D) which was not affected by ropivacaine (NS-R-HTV group; *p* = 1). LPS exposure before HTV ventilation increased MPO activity even more (9-fold over control; *p* < 0.001), which was significantly reduced in mice treated with ropivacaine (*p* = 0.013; Figure 2D).

### Effect of ropivacaine on Src, ICAM-1 and caveolin-1 in lung homogenates of mice challenged with endotoxin alone

**Figure 3:**
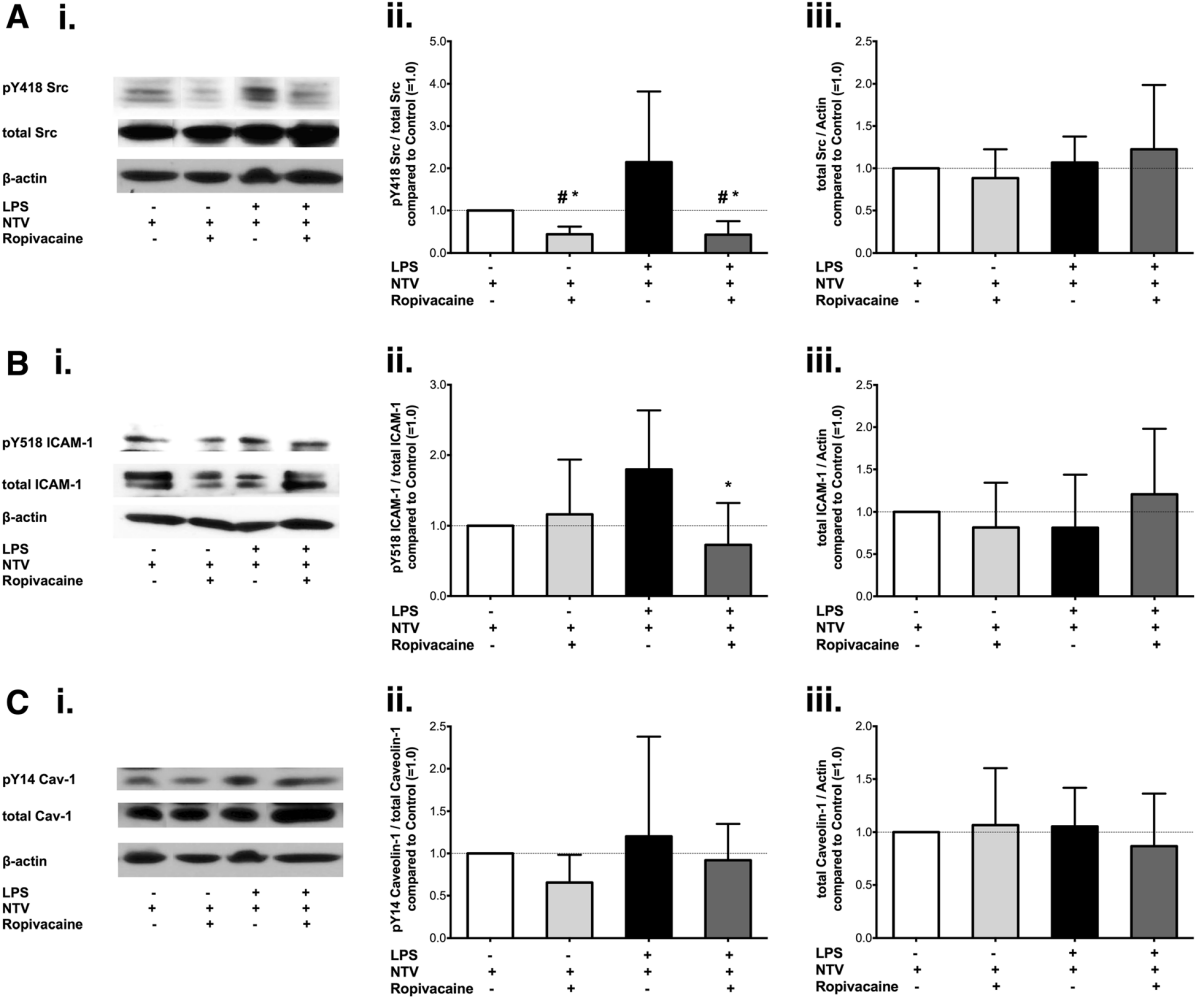
**Effect of ropivacaine on phosphorylation and expression of Src, ICAM-1, and caveolin-1 in the mouse lung during ALI as induced by LPS.** Src protein tyrosine kinase (Src) and its substrates intercellular adhesion molecule-1 (ICAM-1) and caveolin-1 in mouse lung homogenates were assessed after induction of acute lung injury with bacterial lipopolysaccharide (LPS) in C57BL/6 wild type mice. **(A**.**) (i**.**)** Representative Western Blot of lung homogenates probing for Src tyrosine kinase, phosphorylated at tyrosine 418 (pTyr^418^-Src), total Src as well as β-actin. Quantitative densitometry analysis of Western blots showing **(ii**.**)** the ratio of pTyr^418^-Src over total Src compared to control animals (set as 1.0, white bars; N = 5/group), and **(iii**.**)** Src expression as a ratio of total Src over β-actin compared to control animals (set as 1.0, white bars; N = 5/group). **(B**.**) (i**.**)** Representative Western Blot of lung homogenates probing for ICAM-1, phosphorylated at tyrosine 518 (pTyr^518^-ICAM-1), total ICAM-1, as well as β-actin. Quantitative densitometry analysis showing **(ii**.**)** the ratio of pTyr^518^-ICAM-1 over total ICAM-1 compared to control animals (set as 1.0, white bars; N = 5/group), **(iii**.**)** ICAM-1 expression as the ratio of total ICAM-1 over β-actin compared to control animals (set as 1.0, white bars; N = 5/group). **(C**.**) (i**.**)** Representative Western Blot of lung homogenates probing for caveolin-1, phosphorylated at tyrosine 14 (pTyr^14^-Cav-1), total Cav-1 as well as β-actin. Quantitative densitometry analysis showing **(ii**.**)** the ratio of pTyr^14^ Cav-1 over total Cav-1 compared to control animals (set as 1.0, white bars; N = 5/group), and **(iii**.**)** Cav-1 expression as a ratio of total Cav-1 over β-actin compared to control animals (set as 1.0, white bars; N = 5/group). All values shown are mean ± SD; ^#^*p* < 0.05 vs. control (NS-NS-HTV); *p < 0.05 compared to LPS alone.

In order to investigate the anti-inflammatory mechanism by which ropivacaine reduced edema formation (ELW) in mice following treatment with aerosolized LPS, we measured the phosphorylation status and expression levels of Src (Figure 3A), ICAM-1 (Figure 3B) and caveolin-1 (Figure 3C) via Western blot analysis. Src activation, expressed as the ratio of Src, phosphorylated at tyrosine 418, over total Src (Figure 3Aii) was on average 2-fold higher in lungs treated with LPS alone as compared to control animals, however, this effect did not reach statistical significance (n = 5, *p* = 1). Treatment with ropivacaine together with LPS significantly decreased Src activation compared to treatment with LPS alone (*p* = 0.018). There were no differences in Src expression between the groups subjected to NTV ventilation (*p* = 0.64, Figure 3Aiii).

Similar results were obtained for the expression and phosphorylation of ICAM-1 at tyrosine 518, expressed as a ratio of the phosphorylated protein over the total amount of ICAM-1. Expression levels were not altered by any of the treatments prior to NTV ventilation (*p* = 0.451, Figure 3Biii), but there was a non-significant increase of 80% in ICAM-1 phosphorylation after exposure to LPS compared to control (*p* = 0.325) which was completely abolished by ropivacaine (*p* = 0.018, Figure 3Bii).

No significant changes in either caveolin-1 phosphorylation (expressed as the ratio of caveolin-1, phosphorylated at tyrosine 14 over the total amount of caveolin-1, Figure 3Cii, *p* = 0.466) or expression (*p* = 0.874, Figure 3Ciii) were detected (Figures 3Cii and 3Ciii).

### Effect of ropivacaine on Src, ICAM-1 and caveolin-1 in lung homogenates of mice challenged with both endotoxin and hyperinflation

**Figure 4:**
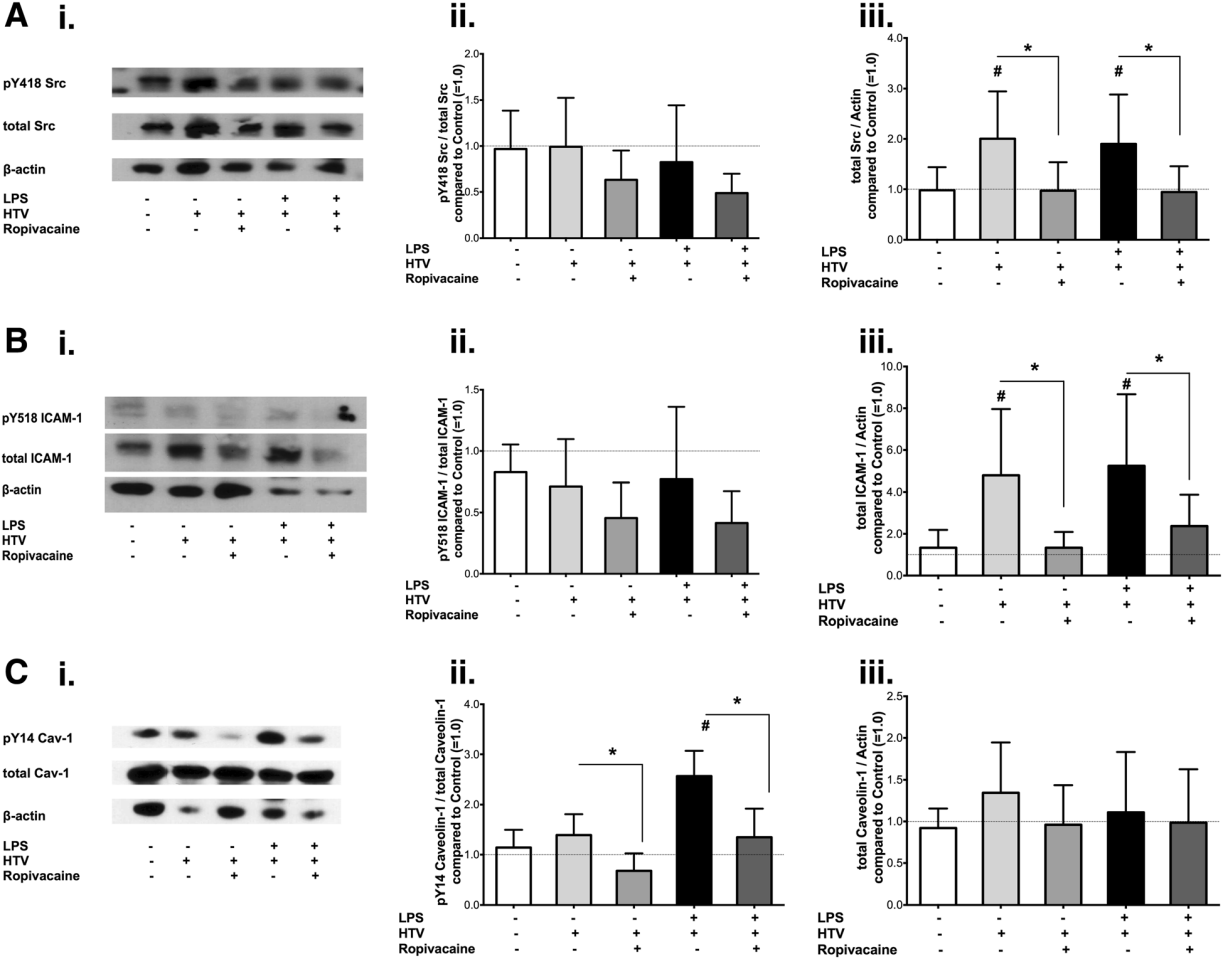
**Effect of ropivacaine on phosphorylation and expression of Src, ICAM-1, and caveolin-1 in the mouse lung during ALI as induced by LPS and hyperinflation (“double-hit” model).** Src protein tyrosine kinase (Src) and its substrates intercellular adhesion molecule-1 (ICAM-1) and caveolin-1 in mouse lung homogenates were assessed after induction of acute lung injury with either hyperinflation (HTV) or a combination of bacterial lipopolysaccharide (LPS) with HTV ventilation (“double-hit” model) in C57BL/6 wild type mice. **(A**.**) (i**.**)** Representative Western Blot of lung homogenates probing for Src tyrosine kinase, phosphorylated at tyrosine 418 (pTyr^418^-Src), total Src as well as β-actin. Quantitative densitometry analysis of Western blots showing **(ii**.**)** the ratio of pTyr^418^-Src over total Src compared to control animals (set as 1.0, white bars; N = 13/group), and **(iii**.**)** Src expression as a ratio of total Src over β-actin compared to control animals (set as 1.0, white bars; N = 13/group). **(B**.**) (i**.**)** Representative Western Blot of lung homogenates probing for ICAM-1, phosphorylated at tyrosine 518 (pTyr^518^-ICAM-1), total ICAM-1, as well as β-actin. Quantitative densitometry analysis showing **(ii**.**)** the ratio of pTyr^518^-ICAM-1 over total ICAM-1 compared to control animals (set as 1.0, white bars; N = 12/group), **(iii**.**)** ICAM-1 expression as the ratio of total ICAM-1 over β-actin compared to control animals (set as 1.0, white bars; N = 12/group). **(C**.**) (i**.**)** Representative Western Blot of lung homogenates probing for caveolin-1, phosphorylated at tyrosine 14 (pTyr^14^-Cav-1), total Cav-1 as well as β-actin. Quantitative densitometry analysis showing **(ii**.**)** the ratio of pTyr^14^ Cav-1 over total Cav-1 compared to control animals (set as 1.0, white bars; N = 10/group), and **(iii**.**)** Cav-1 expression as a ratio of total Cav-1 over β-actin compared to control animals (set as 1.0, white bars; N = 13/group). All values shown are mean ± SD; ^#^*p* < 0.05 vs. control (NS-NS-HTV); *p < 0.05 compared to group as indicated.

To assess the anti-inflammatory mechanism of ropivacaine in the “double-hit” mouse model of ALI, we again measured the phosphorylation status and expression levels of Src, ICAM-1 and caveolin-1 via Western blot analysis in lung homogenates of *WT* mice treated with HTV ventilation +/− LPS and +/− ropivacaine as described above (representative blots shown in Figures 4Ai, 4Bi and 4Ci). Densitometry analysis (n = 12-14) showed no significant changes in Src activation at the 4 hour time point after HTV alone (Figure 4Aii) compared to control (*p* = 1). The application of ropivacaine before HTV ventilation reduced Src-pTyr^418^/Src by 37% compared to HTV alone, however, this effect was not statistically significant (*p* = 0.678). Src expression in lung homogenates (Figure 4Aiii) doubled in both the NS-NS-HTV and the LPS-NS-HTV groups compared to control (*p* = 0.024 and *p* = 0.045, respectively). In both settings – with or without LPS – the addition of ropivacaine completely abolished the observed increase in Src expression (*p* = 0.012 for NS-R-HTV and *p* = 0.026 for LPS-R-HTV; Figure 4Aiii). Similar results were obtained for ICAM-1 phosphorylation (Figure 4Bii); neither HTV ventilation alone nor LPS plus HTV were different from control (*p* = 1 for both comparisons).

Lung tissue ICAM-1 expression (Figure 4Biii), however, increased by 259% compared to control following HTV ventilation (*p* = 0.006) and this increase was completely blocked by ropivacaine (*p* = 0.004). LPS plus HTV ventilation increased ICAM-1 expression to about the same extent as HTV ventilation alone (LPS-NS-HTV, *p* = 0.001 vs. control), which again was reduced by 73% in mice treated with ropivacaine (LPS-R-HTV, *p* = 0.04).

Whereas no effect of ropivacaine was observed on Src and ICAM-1 phosphorylation, caveolin-1 phosphorylation at tyrosine 14 could be blocked by ropivacaine (Figure 4Cii). Ropivacaine plus subsequent HTV ventilation (NS-R-HTV) decreased Cav-1-pTyr^14^/Cav-1 by 51% compared to HTV ventilation alone (*p* < 0.001), and in addition, blocked LPS/HTV-induced increase in Cav-1 phosphorylation (*p* < 0.001; Figure 4Cii). The expression of caveolin-1 (Cav-1) did not change in any of the treatment groups (*p* = 0.501, Figure 4Ciii).

### Requirement of ICAM-1 in mediating acute lung injury in the double-hit mouse model

**Figure 5:**
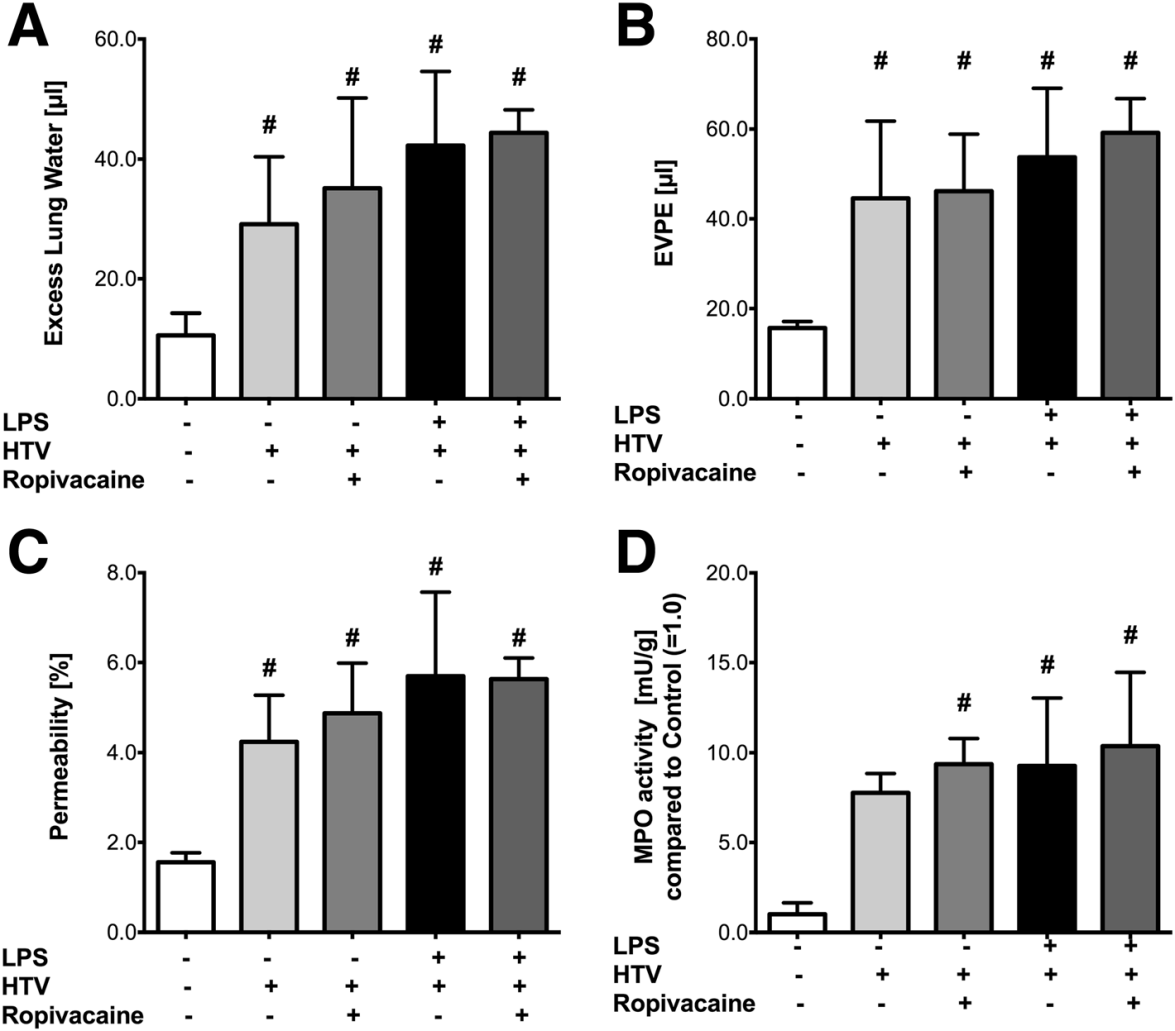
**Effect of ropivacaine on acute lung injury in ICAM-1**^***−/− ***^***mice.*** ALI, induced by either hyperinflation (HTV), bacterial lipopolysaccharide (LPS), or a combination of the two (“double-hit” model) in *ICAM-1*^*−/−*^ mice, was assessed by measuring **(A)** excess lung water (ELW), **(B)** extravascular plasma equivalents (EVPE), **(C)** permeability index, and **(D)** myeloperoxidase activity as described in Methods. Values shown are mean ± SD with n = 7, except for MPO activity (n = 3). ^#^*p* < 0.05 vs. control (NS-NS-HTV); *p < 0.05 vs. LPS-NS-HTV.

To further characterize the mechanism of the anti-inflammatory effect of ropivacaine in the double-hit mouse model of ALI, additional experiments in *ICAM-1*^*−/−*^ mice using the same procedures as described for WT animals were performed. As shown in Figure 5A-D, a significant increase in all measures of ALI (ELW, EVPE, permeability, and MPO activity) induced by HTV ventilation alone in *ICAM-1*^*−/−*^ mice was observed. However, unlike that which was noted in WT mice, LPS did not further increase lung inflammation or permeability, and interestingly, ropivacaine had no effect in *ICAM-1*^*−/−*^ mice. As there was no significant effect of ropivacaine on ALI in *ICAM-1*^*−/−*^ mice, Western blot analyses of the lungs of these animals were not performed.

### Effect of ropivacaine on endotoxin-induced changes in expression/phosphorylation of Src, ICAM-1 and caveolin-1 in cultured human lung microvascular endothelial cells (HLMVECs)

#### Ropivacaine blocks LPS-induced Src activation and expression in HLMVECs

**Figure 6:**
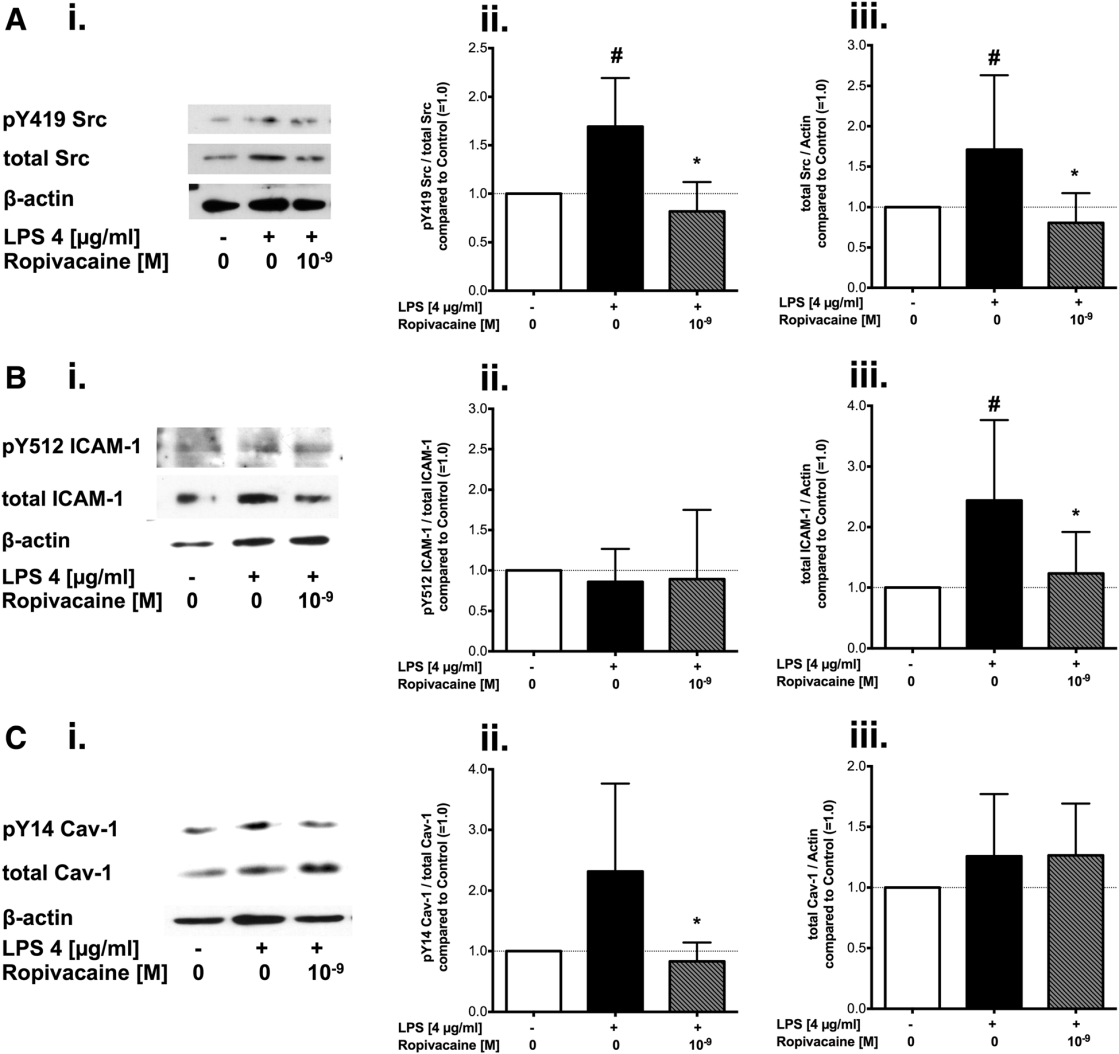
**Effect of ropivacaine on LPS-induced phosphorylation and expression of Src, ICAM-1, and caveolin-1 in cultured endothelial cells.** Representative Western blots and quantitative densitometry analysis of human lung microvascular endothelial cell (HLMVEC) lysates show **(A)** Src, **(B)** ICAM-1, and **(C)** Cav-1 after treatment with LPS (4 μg/ml) in absence or presence of ropivacaine at a concentration of 1 nM (10^−9^ M) for 4 hours. Quantitative densitometry analysis show **(Aii, Bii, Cii)** the ratio of phosphorylated over total protein or **(Aiii, Biii, Ciii)** total protein over β-actin in cells treated with LPS (4 μg/ml) in absence (black bar) or presence (striped grey bar) of ropivacaine compared to untreated cells (white bars, set as 1.0). All values shown are mean ± SD (n = 6-11/group). ^#^*p* < 0.05 vs. untreated cells (control), *p < 0.05 compared to LPS alone.

To determine whether the anti-inflammatory and barrier protective effects of ropivacaine observed in LPS-treated *WT* mouse lungs were due to direct effects of ropivacaine on the endothelium, HLMVEC were incubated with LPS at a concentration of 4 μg/ml for 4 hours in presence or absence of ropivacaine at a concentration of 1 nM (10^−9^ M). Analysis of whole cell lysates was conducted via Western blot, probing for pTyr^419^ Src, total Src, and β-actin (Figure 6Ai). These studies showed that LPS-induced increase in Src activity (69%, *p* = 0.022; Figure 6Aii) and expression (71%, *p* = 0.019, Figure 6Aiii) were completely blocked by 1 nM ropivacaine (*p* = 0.001 for Src activation, Figure 6Aii; *p* < 0.001 for Src expression, Figure 6Aiii).

#### Ropivacaine attenuates LPS-induced ICAM-1 expression in HLMVEC

The same whole cell lysates analyzed via Western Blot for c-Src (~60 kDa) were also analyzed for human ICAM-1 (~120 kDa), phosphorylated at tyrosine 512 (pTyr^512^ ICAM-1), total ICAM-1, and β-actin (Figure 6Bi). ICAM-1 phosphorylation in HLMVEC was not significantly altered by LPS compared to control (*p* = 0.181, Figure 6Bii), whereas ICAM-1 expression increased by 144% after incubation with LPS (*p* = 0.004, Figure 6Biii) and this increase was significantly attenuated in cells co-incubated with ropivacaine (1 nM, *p* = 0.028).

#### Ropivacaine blocks LPS-induced Src-dependent caveolin-1 phosphorylation

HLMVEC lysates incubated with LPS in presence or absence of different concentrations of ropivacaine were analyzed by Western blot, focusing on phosphorylated Cav-1 (pTyr^14^-Cav-1), one of the primary substrates of Src, as well as the total amount of Cav-1 and β-actin (Figure 6 Ci). LPS-induced increase in pTyr^14^-Cav-1 (132% of untreated cells; *p* < 0.001) was completely abolished by 1 nM ropivacaine (*p* = 0.003, Figure 6Cii). Neither incubation of cells with different concentrations of ropivacaine alone (*p* = 0.411) nor with LPS (*p* = 1) significantly altered total Cav-1 expression compared to untreated cells (Figure 6Ciii).

### Active Src is required for endotoxin-induced increase in ICAM-1 expression

**Figure 7:**
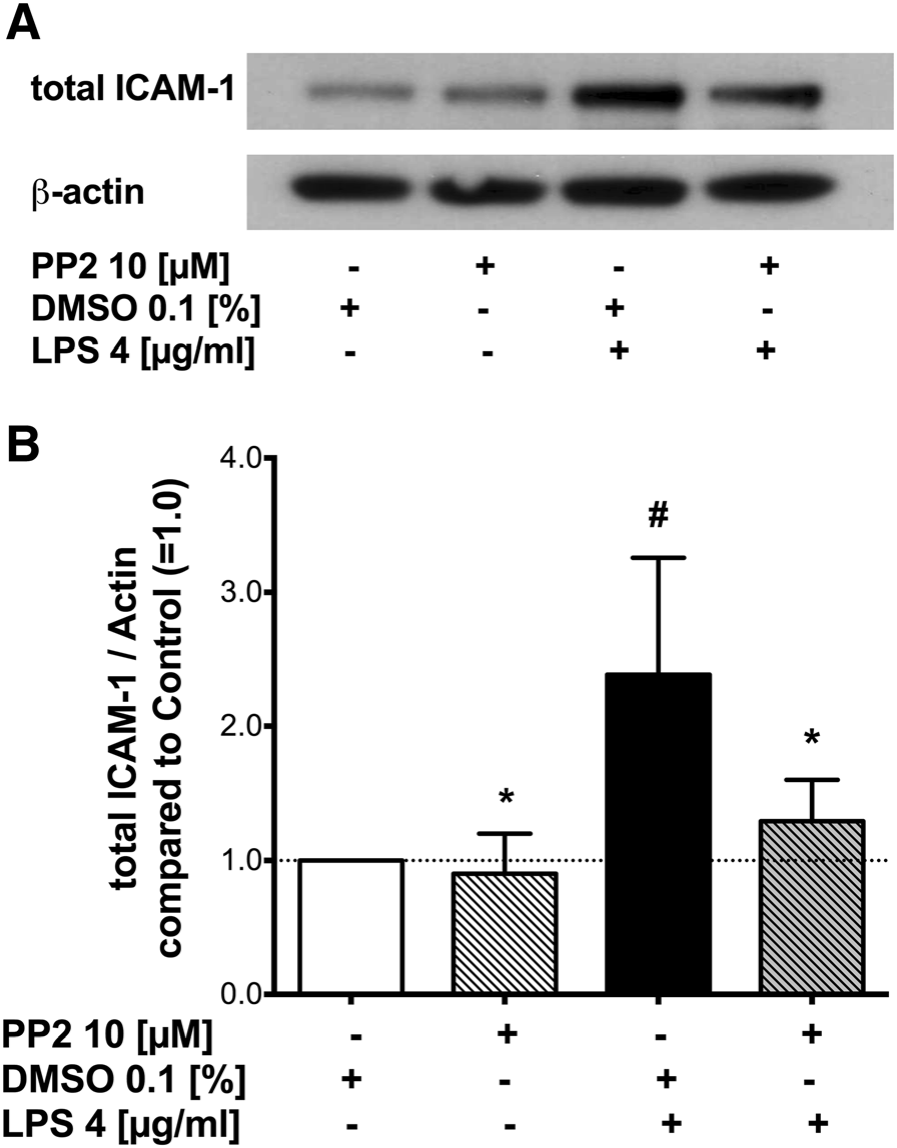
**Effect of Src inhibitor on LPS-induced ICAM-1 expression in HLMVEC. (A)** Representative Western blot of HLMVEC ICAM-1 (total ICAM-1, row 1) and β-actin (row 2) after treatment of the cells with either vehicle (dimethylsulfoxide, DMSO 0.1%), Src inhibitor PP2 (pretreatment for 30 minutes at a concentration of 10 μM), LPS (4 μg/ml), or LPS following pretreatment with PP2. **(B)** Quantitative densitometry analysis of the ratio of total ICAM-1 over β-actin. Values shown are mean ± SEM. ^#^*p* < 0.05 vs. vehicle (control), *p < 0.05 compared to LPS alone.

To evaluate whether Src activation is required for the observed increase in ICAM-1 expression induced by LPS, we incubated HLMVEC with LPS in absence or presence of Src kinase inhibitor PP2 shown previously to effectively block Src activation [13] (Figure 7A). Densitometry analysis (n = 6) confirmed that LPS induced a 139% increase in ICAM-1 expression compared to cells treated with vehicle alone (DMSO). PP2 blocked the LPS-induced increase in ICAM-1 expression by 79% (*p* = 0.005), but had no effect on ICAM-1 expression in absence of LPS (*p* = 1; Figure 7B) indicating LPS-induced increase in ICAM-1 is mediated in part via Src signaling.

## Discussion

The objective of this study was to determine the anti-inflammatory mechanism of action of the local anesthetic ropivacaine using both *in vivo* and *in vitro* approaches. We assessed ALI induced by LPS exposure or HTV ventilation alone and in combination (i.e., “double-hit” model). The combination of the two stimuli increases the severity of ALI [6], most likely due to additive effects of mechanical injury and inflammatory cytokine signaling [6,28].

The present study demonstrated that low-dose ropivacaine can attenuate ALI induced by LPS or the “double-hit” of LPS and HTV, but not by HTV ventilation alone. We also showed that this observed decrease might be due to an inhibition of LPS-induced Src activation/expression, ICAM-1 expression, and Cav-1 phosphorylation. Our primary objective was to test the effect of the local anesthetic ropivacaine on HTV ventilation alone or HTV in combination with bacterial endotoxin as it was shown previously [9] that ALI induced by “high dose” LPS (delivered intraperitoneally or intratracheally) could be attenuated by ropivacaine. Changes in vascular permeability were rather small 4 hours after “low dose” LPS exposure, which were comparable to those induced by mechanical ventilation alone [6]. Thus, previous results were confirmed by our study.

The combination of two harmful stimuli, LPS and HTV ventilation, can exponentially increase the severity of ALI [6], most likely due to additive effects of mechanical injury and inflammatory cytokine signaling [6,28], which may be relevant to mechanically-ventilated patients in the intensive care unit setting [29]. We observed a protective effect of ropivacaine only in animals treated with both stimuli or with LPS alone, whereas there was no effect of ropivacaine in animals challenged by HTV ventilation alone, indicating that ropivacaine selectively inhibits inflammatory signaling induced by LPS in in the lung – independent of ventilation.

The dose of intravenous ropivacaine used in our experimental setting (0.33 mg/kg or 0.01 mg for a 30 g mouse) was chosen based on previous experiments where this dose was shown to be effective in attenuating LPS-induced lung injury [9]. The conversion factor for determining what the ropivacaine dose in mice would equate to in humans is approximately 12:1 [30,31], which means our animal dose would be equivalent to a human dose of 0.027 mg/kg, or a total of 1.89 mg for a 70 kg patient. Mean (total) plasma concentration of ropivacaine of 1.82 mg/l (5.5 μM) has been reported to be present (and well-tolerated) in patients after continuous epidural infusions of 0.2% ropivacaine at a rate of 10 mg/hour for 48 hours after major abdominal or urologic surgery [32], thus indicating the ropivacaine dose used in our study is clinically relevant. Additionally, the concentrations used in cell culture experiments were previously proven to have no effect on the viability of rat lung epithelial and endothelial cells [9] as well as human lung adenocarcinoma cells [26].

Multiple mechanisms facilitate the transition from the initial “barotrauma” due to high inspiratory pressures to the distinct “biotrauma” of VILI [33,34]. Cyclic stretch of the lung, as in HTV ventilation, leads to a severe physical injury [33], but at the same time induces inflammatory signaling locally in alveolar epithelial cells, macrophages, and neutrophils which together with the direct effect of stretch on the endothelium, leads to barrier disruption, pulmonary edema, and neutrophil infiltration into alveolae [4,34]. The results of the present study suggest that ropivacaine partially blocked inflammatory signaling induced by HTV ventilation, but not the physical injury to the lung induced by stretch. Although ventilator- and LPS-induced lung injury share common signaling pathways, e.g. Src-dependent caveolin-1 and ICAM-1 signaling[6,13,19,20,25,35-38], the physical injury induced by HTV ventilation is much more severe and includes mechanical disruption of cell membranes and subsequent necrosis[33,39]. Presumably 60% of the injured cells are able to repair their membrane defects, a process that also involves inflammatory signaling via the NFκB pathway [33,40]. However, the severity and complexity of the stretch-induced injury might explain the differences observed between Western blots, where activation of inflammatory Src signaling *in vivo* and *in vitro* were shown to be attenuated by ropivacaine, and *in vivo* measures of ALI/VILI, where ropivacaine had no effect on VILI *per se*. Additionally, ropivacaine was applied intravenously through a central venous catheter and therefore the effects were primarily exerted on endothelial cells. Thus, subsequent *in vitro* experiments in this study using human lung microvascular endothelial cells focused on establishing a molecular mechanism by which ropivacaine exerts its protective effects by blocking the signaling events initiated by LPS.

Endothelial hyperpermeability due to barrier dysfunction of pulmonary capillaries is a key element of the pathogenesis of ALI and ARDS [1]. It was shown previously [9] that ALI induced by “high dose” LPS (delivered intraperitoneally or intratracheally) could be attenuated by ropivacaine. Our results indicated that ropivacaine was only able to attenuate ALI induced by HTV + LPS, having no effect on HTV alone. To gain novel mechanistic insight into the proposed anti-inflammatory properties of ropivacaine on inflammation-induced endothelial barrier dysfunction and lung edema [41], we assessed LPS-induced inflammatory Src, ICAM-1, and caveolin-1 (Cav-1) signaling in human lung microvascular endothelial cells (HLMVECs) *in vitro*. The observed inhibition of LPS-induced expression/activation of Src, ICAM-1, and Cav-1 in endothelial cells by ropivacaine provided significant insight into the possible anti-inflammatory mechanism and potential therapeutic benefit of amide-linked local anesthetics on the pathogenesis of ALI.

We, and others, have shown that Src protein tyrosine kinase (Src) is a key regulator of endothelial barrier function. Src-dependent phosphorylation of ICAM-1 tyrosine 512 (human, tyrosine 518 in mice) increases neutrophil adhesion that can give rise to vascular inflammation [14] and Cav-1-dependent pulmonary vascular leakage [35]. Additionally, Src phosphorylation of vascular endothelial (VE-) cadherin on two distinct tyrosine residues (Y658 and Y685) is thought to regulate adherens junction stability, ultimately resulting in increased vascular permeability [42-44]. It was also demonstrated that ropivacaine and lidocaine inhibit inflammatory endothelial cell Src activation and signaling induced by TNFα by preventing the initial signaling step downstream of TNF receptor-1, thus preventing endothelial barrier disruption as well as neutrophil adhesion [45].

In the current study, we showed that low-dose ropivacaine (1 nM *in vitro* and 0.01 mg *in vivo*) lead to a reduction in LPS-induced Src expression and activation. However, we did not observe a significant difference in LPS-induced Src activity in the animals challenged by both LPS and hyperinflation, but rather only in animals exposed to LPS alone. This might be explained by the fact that the total lung homogenate contains several cell types (epithelial, endothelial, smooth muscle, fibroblasts, macrophages etc.) and that each of these cell types may react differently to LPS, HTV ventilation (and the combination of the two), and ropivacaine in terms of Src activation.

ICAM-1, a cell surface glycoprotein belonging to the immunoglobulin superfamily of adhesion molecules, is expressed in several different cell types including lung endothelial [16] and epithelial cells [46]. ICAM-1 is required for firm adhesion of neutrophils to the endothelium and for initiation of transendothelial neutrophil migration during vascular and systemic inflammation [16]. Additional experiments with *ICAM-1*^*−/−*^ mice in the present study confirmed that LPS-induced ALI in animals ventilated with HTV is clearly ICAM-1-dependent, whereas the lung injury caused by HTV alone might (at least in part) be independent of ICAM-1 and reflect the physical cell damage caused by cyclic stretch [33,39,40]. LPS activation of toll-like receptor 4 [6] promotes the release of inflammatory cytokines which recruit neutrophils to vascular, interstitial, and alveolar spaces of the lung [47], thereby leading to inflammatory hyper-permeability via disruption of the endothelial barrier [35,48].

Interestingly, this investigation also demonstrated that Src activation is required for the observed increase in ICAM-1 expression induced by LPS in endothelial cells. Pre-treatment of cells with the Src inhibitor PP2 significantly blocked the increase in ICAM-1 expression, and thus together with the known requirement of nuclear factor κB (NF-κB) activation in the mechanism of LPS-induced increase in ICAM-1 expression [49], Src signaling also plays a primary role. Consistent with the hypothesis that Src signaling upstream of NF-κB mediates LPS-induced ICAM-1 expression, Src activation of NF-κB was demonstrated in macrophages stimulated with LPS [50] and in pancreatic acinar cells stimulated with caerulein [51].

Caveolin-1 is required for formation and trafficking of caveolae, the primary vesicular carrier and mechanism of transcellular macromolecule transport (e.g. albumin) through the vascular endothelial barrier [52,53]. Increasingly, evidence points to the role of Cav-1 and caveolae-mediated transcellular albumin transport (transcytosis) in the pathogenesis of ALI in general [12,18-20,28,37,54] and VILI in particular [20], although it could also be shown that caveolin-1 deficient mice developed a more severe VILI than WT animals [22]. In line with the current study, we previously showed that Src phosphorylation of Cav-1 tyrosine 14 [12] contributes to the development of vascular hyperpermeability via paracellular and transcellular pathways [19]. Here, we demonstrated that the inflammatory signaling mechanisms that promote Cav-1 phosphorylation-dependent increase in transcellular as well as paracellular permeability during VILI [20,55] were blocked by ropivacaine. We observed a reduction in both Src activation and Cav-1 phosphorylation induced by HTV ventilation in animals treated with ropivacaine, although this did not abolish HTV (alone) induced ALI/VILI.

## Conclusion

Low dose ropivacaine attenuated ALI induced by LPS alone as well as in the “double-hit” mouse model induced by the combination of LPS and hyperinflation, whereas there was no effect of ropivacaine on VILI induced by HTV ventilation alone or on ALI induced by the double-hit protocol in *ICAM-1*^*−/−*^ mice. This protective effect of ropivacaine was associated with a decrease in LPS-induced Src activation, ICAM-1 expression, and caveolin-1 phosphorylation suggesting that ropivacaine may have significant therapeutic potential for treating pulmonary vascular inflammation and endothelial hyperpermeabilty, thus preventing or tempering the development of ALI.

## Competing interest

The authors report no conflict of interest.

## Authors’ contributions

TP helped conceive the study, conducted experiments, analyzed the data, and wrote the manuscript; ROD helped with data analysis and wrote the manuscript, GH helped conceive the study and conducted experiments; MC and AZC conducted experiments; EGV helped conceive the study and wrote the manuscript; AB helped conceive the study and wrote the manuscript; DES helped conceive the study and wrote the manuscript; BBS helped conceive the study, performed data analysis, and wrote the manuscript; RDM conceived the study, analyzed data, and wrote the manuscript. The manuscript was read and approved by all the authors.

## Pre-publication history

The pre-publication history for this paper can be accessed here:

http://www.biomedcentral.com/1471-2253/14/57/prepub
